# Supplementary material for: Effects of Maternal Gut Microbiota-Targeted Therapy on the Programming of Nonalcoholic Fatty Liver Disease in Dams and Fetuses, Related to a Prenatal High-Fat Diet
Source: Nutrients. 2022 Sep 27;14(19):4004. doi: 10.3390/nu14194004 (PMC9573493; doi:10.3390/nu14194004)
Supplement: Supplementary file 1 [file nutrients-14-04004-s001.zip › Supplementary Tables(1).pdf]

Supplementary Table S1. Comparison for the control chow diet and high fat diet with the feed ingredients

| | Control chow diet | High-fat diet |
|---|---|---|
| Carbohydrates (g/100g) | 67.3 | 35.5 |
| Protein (g/100g) | 19.2 | 23.0 |
| Fat (g/100g) | 4.3 | 35.8 |
| Energy (kcal/gm) | 3.85 | 5.56 |

D12331 Rodent Diet With 58 kcal% Fat and Sucrose

| Class description | Ingredients | Grams |
|---|---|---|
| Protein | Casein, Lactic, 30 Mesh | 228.00 g |
| Protein | Methionine, DL | 2.00 g |
| Carbohydrate | Sucrose, Fine Granulated | 184.00 g |
| Carbohydrate | Lodex 10 | 170.00 g |
| Fat | Coconut Oil, 101 | 333.50 g |
| Fat | Soybean Oil, USP | 25.00 g |
| Mineral | S10001A | 20.00 g |
| Mineral | Calcium Phosphate, Dibasic | 20.00 g |
| Mineral | Sodium Bicarbonate | 10.50 g |
| Mineral | Potassium Citrate, Monohydrate | 4.00 g |
| Vitamin | Choline Bitartrate | 2.00 g |
| Vitamin | V10001C | 1.00 g |
| Dye | Dye, Red FD&C #40, Alum. Lake 35-42% | 0.10 g |

Supplementary Table S2. The primer sequences of mRNA used in this study.

| Rat Gene | primer | sequence (5' to 3') |
|---|---|---|
| *ACC1* | Forward | TGAGGAGGACCGCATTTATC |
| | Reverse | GCATGGAATGGCAGTAAGGT |
| *FAS* | Forward | AGATCCTGGAACGTGAACATGA |
| | Reverse | GCCGTACTTCACGAATGGGT |
| *LPL* | Forward | GTACAGTCTTGGAGCCCATGC |
| | Reverse | GCCAGTAATTCTATTGACCTTCTTGTT |
| *SIRT1* | Forward | TGTTTCCTGTGGGATACCTGA |
| | Reverse | TGAAGAATGGTCTTGGGTCTTT |
| *PPARα* | Forward | TTCATCACCCGAGAGTTCCTA |
| | Reverse | CCGATCTCCACAGCAAATTAT |
| *PPARγ* | Forward | CTTTATGGAGCCTAAGTTTGAGT |
| | Reverse | GTTGTCTTGGATGTCCTCG |
| *mTOR* | Forward | TTGAGGTTGCTATGACCAGAGAGAA |
| | Reverse | TTACCAGAAAGGACACCAGCCAATG |
| *AMPKα2* | Forward | GTCGGCACCTTCGGCAAAGTGAA |
| | Reverse | AGAAATTCACCATCTGACATCATATTAGA |
| *PGC1α* | Forward | AGGTCCCCAGGCAGTAGAT |
| | Reverse | CGTGCTCATTGGCTTCATA |
| *GPR41* | Forward | TGACGGTGAGCATAGAACGTTT |
| | Reverse | GCCGGGTTTTGTACCACAGT |
| *GPR43* | Forward | CACCGAGAACCAAATCACCT |
| | Reverse | GAGGGACTCTGCCTCAAGTG |
| *GPR120* | Forward | CCAACCGCATAGGAGAAATC |
| | Reverse | CAAGCTCAGCGTAAGCCTCT |
| *18S* | Forward | GCGATGCGGCGGCGTTAT |
| | Reverse | AGACTTTGGTTTCCCGGAAGC |
| *GAPDH* | Forward | TCTTGTGCAGTGCCAGCCTC |
| | Reverse | GTCACAAGAGAAGGCAGCCCTGG |

Abbreviation: ACC1, acetyl-CoA carboxylase 1; FAS, fatty acid synthetase; LPL, lipoprotein lipase; SIRT1, Sirtuin 1; PPARα, Peroxisome proliferator-activated receptor α; PPARγ, Peroxisome proliferator-activated receptor γ; mTOR, mammalian target of rapamycin; AMPKα2, AMP-activated protein kinase; PGC1α, peroxisome proliferator-activated receptor gamma coactivator 1α; GPR41, G-protein-coupled receptor 41; GPR43, G-protein-coupled receptor 43; OLFR59, olfactory receptor 59; GAPDH, glyceraldehyde 3-phosphate dehydrogenase

Supplementary Table S3. The weekly body weight change of study animals

| | 8w | 9w | 10w | 11w | 12w | 13w |
|---|---|---|---|---|---|---|
| CC | 212.40±3.74 | 210.28±3.06 | 216.00±3.14 | 229.44±3.02 | 232.81±3.08 | 241.79±3.23 |
| HF | 216.49±4.74 | 234.30±6.45* | 255.59±9.52* | 273.39±10.33* | 288.81±11.33* | 298.59±11.38* |
| H+L | 217.24±4.27 | 232.88±4.75* | 253.10±6.39* | 268.18±7.72* | 277.62±9.12* | 287.34±9.20* |
| H+B | 209.78±3.27 | 226.87±3.14* | 249.00±3.61* | 266.85±4.31* | 271.97±3.38* | 284.33±1.91* |

| | 14w | 15w | 16w | 17w | 18w | 19w |
|---|---|---|---|---|---|---|
| CC | 254.09±3.53 | 245.25±5.04 | 251.50±4.59 | 273.05±4.67 | 287.13±5.34 | 318.22±4.46 |
| HF | 308.63±11.89* | 307.99±13.10* | 312.17±14.85* | 318.94±21.01* | 358.26±12.86* | 399.47±11.65* |
| H+L | 295.77±9.66* | 292.30±9.19* | 291.30±9.78* | 304.63±9.11* | 325.41±11.61*# | 355.76±12.81*# |
| H+B | 294.00±5.08* | 294.83±4.41* | 294.33±5.05* | 317.15±4.98* | 339.65±6.18* | 377.83±11.82* |

The results are presented as mean ± standard error.; *compared with CC, $P$ <0.05;

# compared with HF, $P$ <0.05.

Abbreviation: CC, maternal control diet; HF. maternal high-fat diet; H+L, maternal high-fat diet plus *Lactobacillus reuteri* treatment; H+B, maternal high-fat diet plus sodium butyrate treatment

Supplementary Table S4. The biochemical markers of dams

| | CC | HF | B | H+B |
|---|---|---|---|---|
| AST | 85.90 ± 9.32 | 105.25 ± 12.07 | 77.00 ± 4.77 | 80.33 ± 7.16 |
| ALT | 34.30 ± 1.13 | 37.00 ± 2.46 | 57.75 ± 11.60$^{*\#}$ | 36.00 ± 2.67$^{\$}$ |
| T-Cholesterol | 61.70 ± 5.39 | 67.00 ± 7.70 | 56.00 ± 7.16 | 51.83 ± 3.18 |
| HDL | 36.40 ± 3.52 | 53.25 ± 6.08 | 34.75 ± 3.57$^{\#}$ | 42.17 ± 3.15 |

| | CC | HF | L | H+L |
|---|---|---|---|---|
| AST | 85.90 ± 9.32 | 105.25 ± 12.07 | 86.43 ± 8.32 | 102.40 ± 10.04 |
| ALT | 34.30 ± 1.13 | 37.00 ± 2.46 | 40.14 ± 1.87 | 35.40 ± 3.14 |
| T-Cholesterol | 61.70 ± 5.39 | 67.00 ± 7.70 | 64.71 ± 3.32 | 49.70 ± 6.00$^{\#}$ |
| HDL | 36.40 ± 3.52 | 53.25 ± 6.08 | 39.14 ± 3.94 | 36.60 ± 4.79$^{\#}$ |

The results are presented as mean ± standard error; *compared with CC, *P* <0.05;

$^{\#}$ compared with HF, *P* <0.05, $^{\$}$ as compared with B, *P* <0.05

Abbreviation: CC, maternal control diet; HF. maternal high-fat diet; B, maternal control diet plus sodium butyrate treatment; H+B, maternal high-fat diet plus sodium butyrate treatment; L, maternal control diet plus *Lactobacillus reuteri* treatment; H+L, maternal high-fat diet plus *Lactobacillus reuteri* treatment; AST, aspartate aminotransferase; ALT, aspartate aminotransferase; T-Cholesterol, total cholesterol; HDL, high-density lipoprotein
