# Supplementary figures and images for: Effects of Maternal Gut Microbiota-Targeted Therapy on the Programming of Nonalcoholic Fatty Liver Disease in Dams and Fetuses, Related to a Prenatal High-Fat Diet
Source: Nutrients. 2022 Sep 27;14(19):4004. doi: 10.3390/nu14194004 (PMC9573493; doi:10.3390/nu14194004)

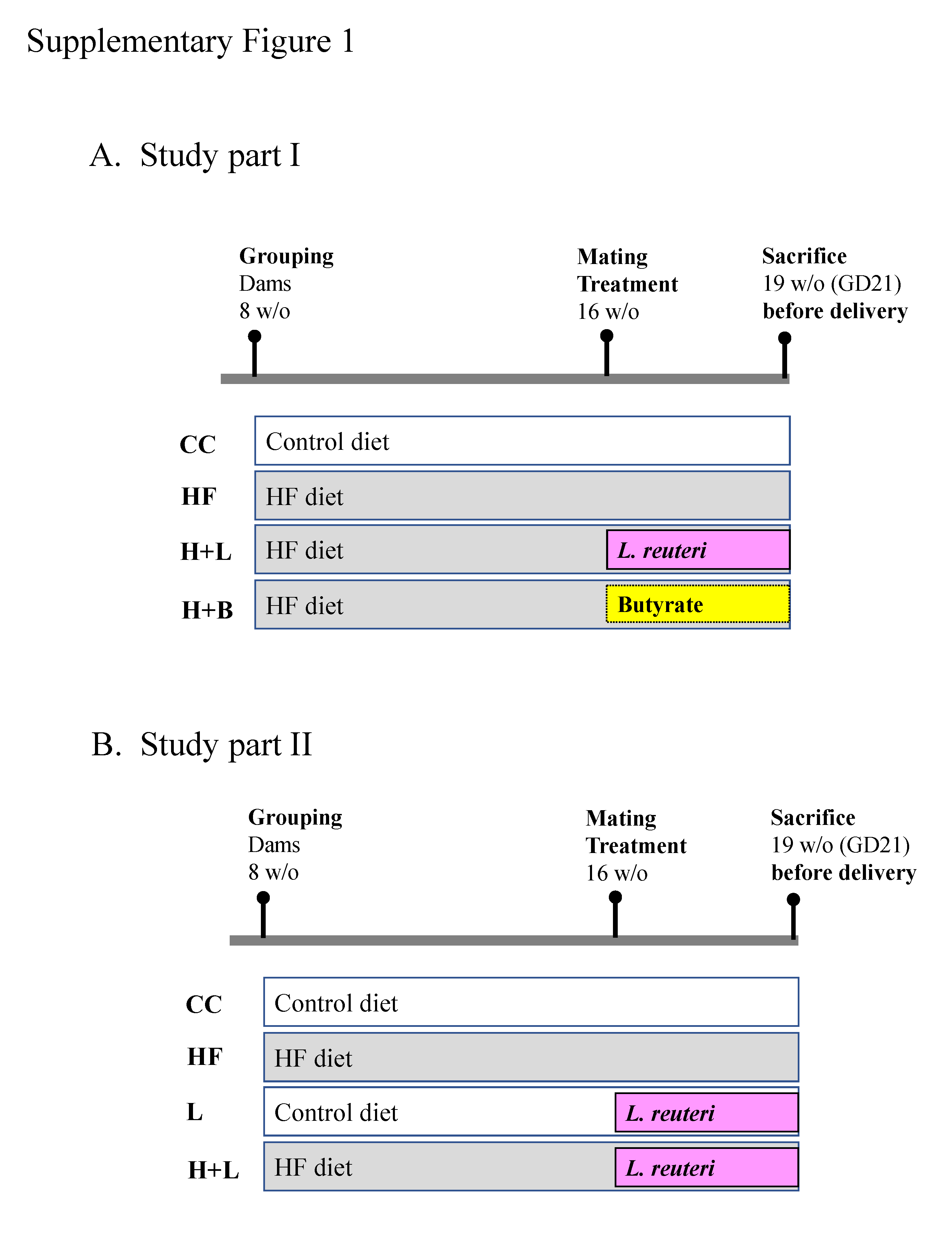

Supplement: Supplementary file 1 [file nutrients-14-04004-s001.zip › supplementary figure S1.tif]

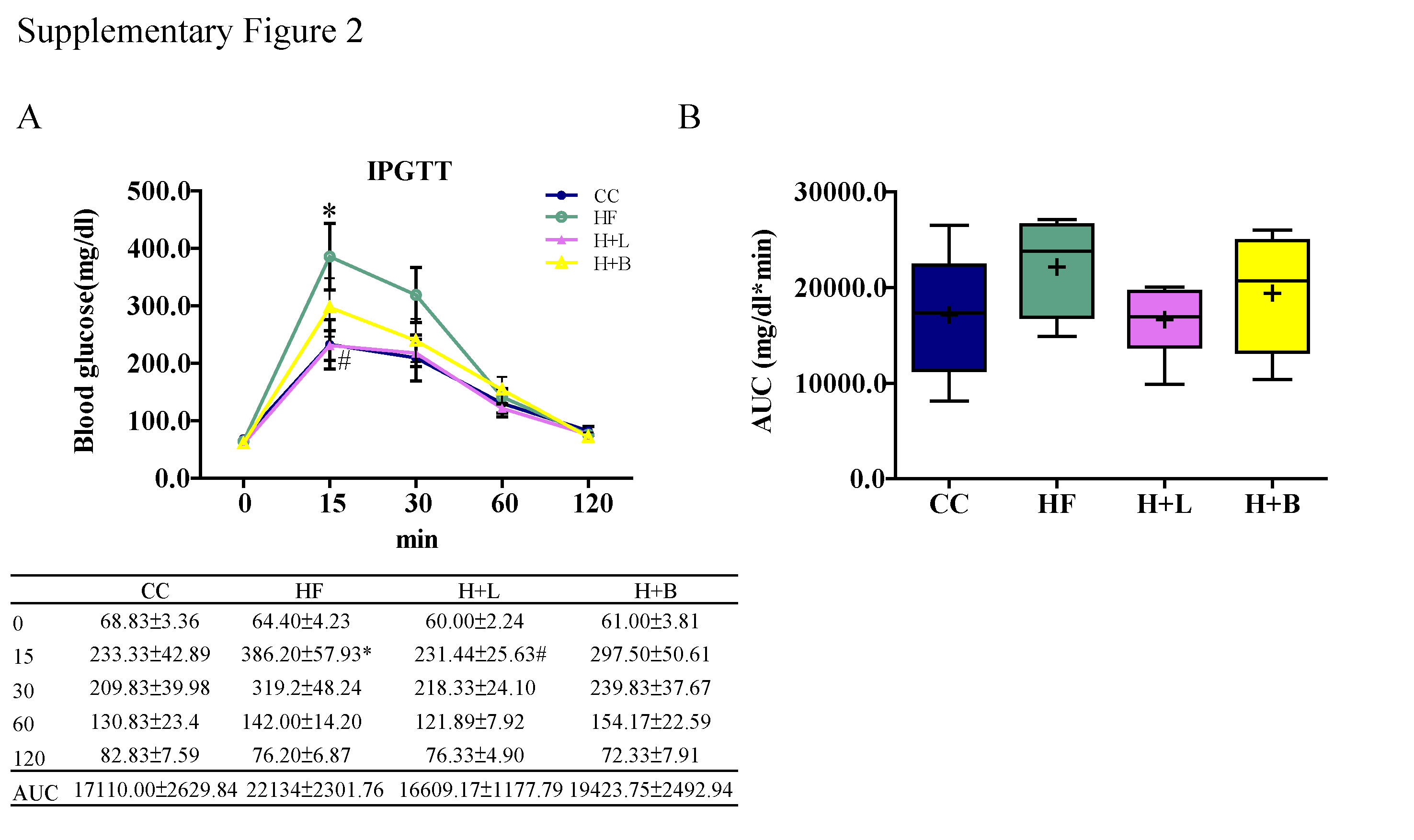

Supplement: Supplementary file 1 [file nutrients-14-04004-s001.zip › supplementary figure S2.tif]

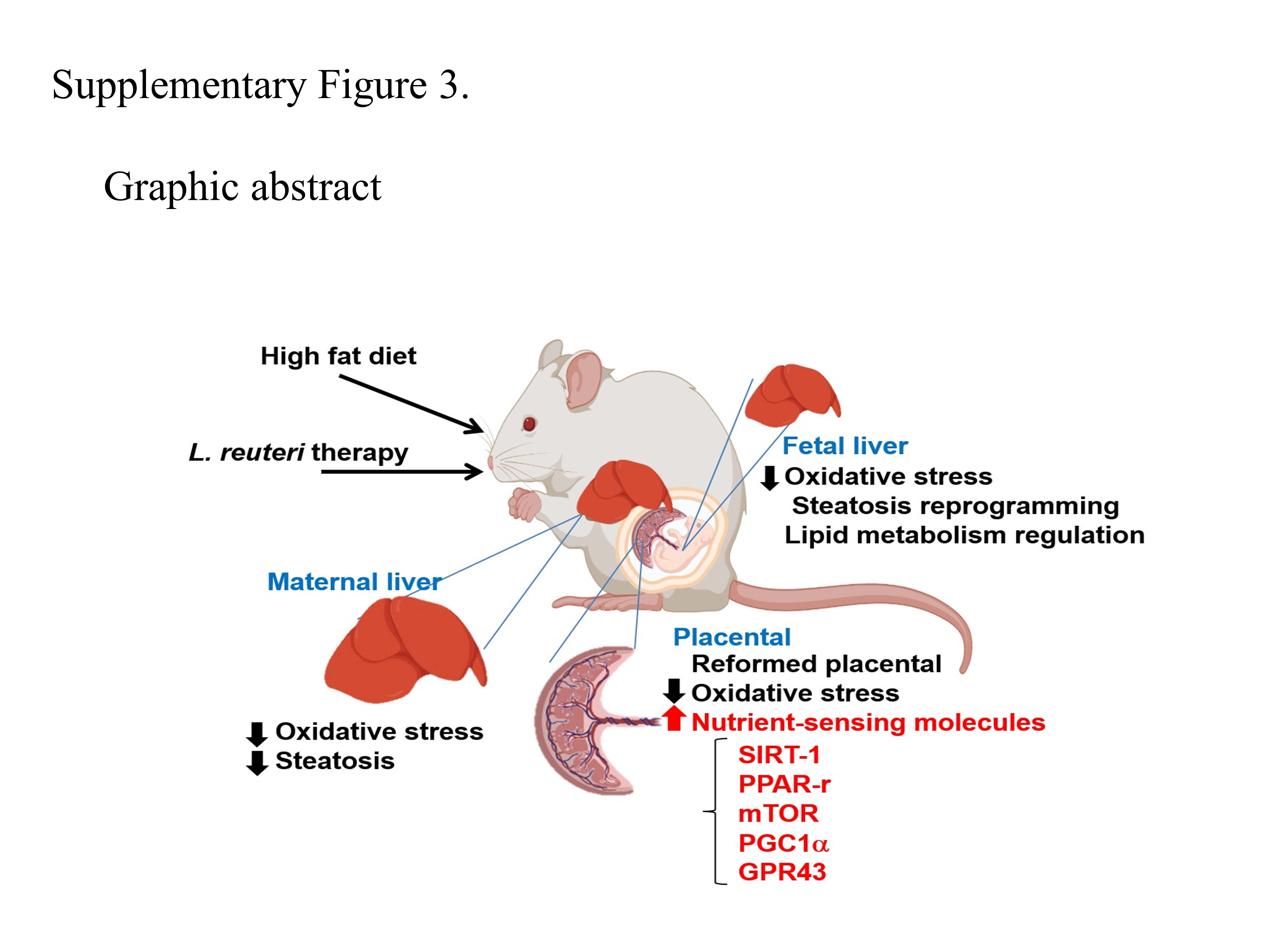

Supplement: Supplementary file 1 [file nutrients-14-04004-s001.zip › supplementary figure S3.tif]
